# 6-Bromo-2-(diprop-2-ynyl­amino)-1*H*-benzo[*de*]isoquinoline-1,3(2*H*)-dione

**DOI:** 10.1107/S1600536812022507

**Published:** 2012-05-23

**Authors:** Jing-Song Lv, Jun-Lei Wang, Cheng-He Zhou

**Affiliations:** a Laboratory of Bioorganic & Medicinal Chemistry, School of Chemistry and Chemical Engineering, Southwest University, Chongqing 400715, People’s Republic of China; b School of Chemistry and Chemical Engineering, Bijie University, Bijie, Guizhou 551700, People’s Republic of China

## Abstract

The asymmetric unit of the title compound, C_18_H_11_BrN_2_O_2_, contains two independent mol­ecules in which the prop-2-ynyl­amino groups have different mutual orientations. In one mol­ecule, the Br atom is disordered over two positions, with refined occupancies of 0.742 (2) and 0.258 (2).

## Related literature

For the applications and biological activity of naphthalimide compounds, see: Muth *et al.* (2007 ▶); Zhang & Zhou (2011 ▶); Zhang *et al.* (2011 ▶). For the synthesis, see: Wang *et al.* (2010 ▶).
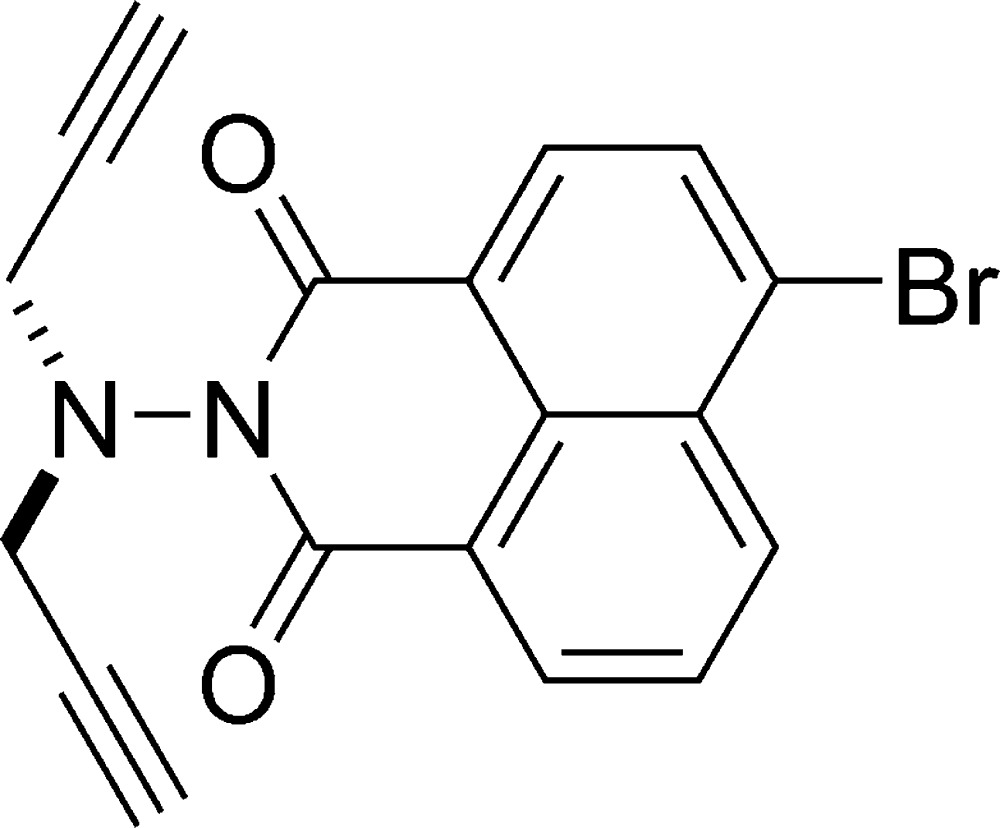


## Experimental

### 

#### Crystal data

C_18_H_11_BrN_2_O_2_
*M* *_r_* = 367.20
Triclinic, 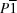

*a* = 10.460 (3) Å
*b* = 11.816 (3) Å
*c* = 13.561 (3) Å
α = 75.128 (4)°
β = 75.725 (4)°
γ = 72.298 (4)°
*V* = 1517.2 (7) Å^3^
*Z* = 4
Mo *K*α radiation
μ = 2.72 mm^−1^
*T* = 296 K
0.26 × 0.24 × 0.18 mm

#### Data collection

Bruker APEXII CCD diffractometer
Absorption correction: multi-scan (*SADABS*; Sheldrick, 1996 ▶) *T*_min_ = 0.538, *T*_max_ = 0.640
8441 measured reflections
5901 independent reflections
3709 reflections with *I* > 2σ(*I*)
*R*_int_ = 0.023

#### Refinement

*R*[*F* ^2^ > 2σ(*F* ^2^)] = 0.067
*wR*(*F* ^2^) = 0.189
*S* = 1.04
5901 reflections
425 parameters
1 restraint
H-atom parameters constrained
Δρ_max_ = 2.20 e Å^−3^
Δρ_min_ = −0.74 e Å^−3^

### 

Data collection: *APEX2* (Bruker, 2001 ▶); cell refinement: *SAINT* (Bruker, 2001 ▶); data reduction: *SAINT*; program(s) used to solve structure: *SHELXS97* (Sheldrick, 2008 ▶); program(s) used to refine structure: *SHELXL97* (Sheldrick, 2008 ▶); molecular graphics: *PLATON* (Spek, 2009 ▶); software used to prepare material for publication: *SHELXTL* (Sheldrick, 2008 ▶).

## Supplementary Material

Crystal structure: contains datablock(s) global, I. DOI: 10.1107/S1600536812022507/lh5464sup1.cif

Structure factors: contains datablock(s) I. DOI: 10.1107/S1600536812022507/lh5464Isup2.hkl

Supplementary material file. DOI: 10.1107/S1600536812022507/lh5464Isup3.cml

Additional supplementary materials:  crystallographic information; 3D view; checkCIF report

## Figures and Tables

**Fig. 1.**
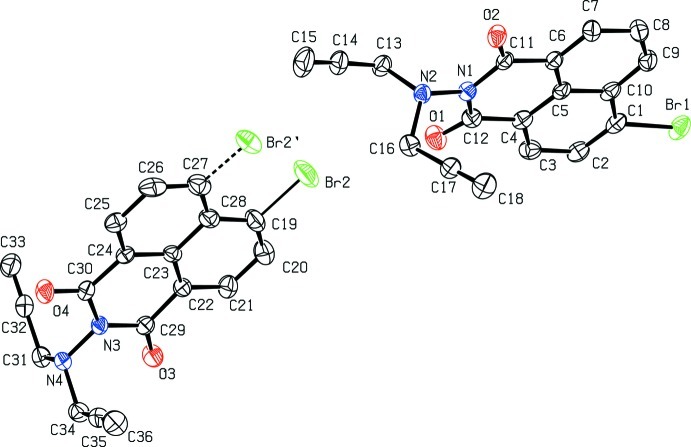
The asymmetric unit of (I), showing displacement ellipsoids are drawn at the 30% probability level. The minor component of disorder is shown with a dashed bond.

## supplementary crystallographic information

### Comment

Naphthalimide compounds are an important type of cyclic imides with strong hydrophobicity and a desirable large π-conjugated backbone. This special aromatic heterocycle can easily interact with various active targets in biological systems *via* non-covalent forces such as π···π stacking (Muth *et al.*, 2007), and exhibit diverse biological activities including anticancer, antibacterial, antitrypanosomal, analgesic, photobiological and antinociceptive potency (Zhang & Zhou, 2011). Our interest is to develop novel naphthalimide-derived compounds as potential antimicrobial agents (Zhang *et al.*, 2011). Herein, the crystal structure of the title compound (I) is reported.

The asymmetric unit of (I) is shown in Fig. 1. In the two independent molecules, the prop-2-ynylamino groups have different mutual orientations (see Fig. 1). In one moeclue the Br atom is disordered over two positions with refined occupancies 0.742 (2) and 0.258 (2).

### Experimental

Compound (I) was prepared according to the procedure of Wang *et al.* (2010). A mixture of 2-amino-6-bromo-1*H*-benzo[de]isoquinoline-1,3(2*H*)-dione (1.00 g, 3.44 mmol), anhydrous potassium carbonate (1.43 g, 10.31 mmol) and propargyl bromide (1.02 g, 8.59 mmol) in *N*, *N*-dimethylacetamide (20 ml) was stirred at 353K. After the reaction was complete (monitored by TLC, petroleum ether/ethyl acetate, 3/1, V/V), the solvent was removed. The residue was dissolved in dichloromethane (30 ml) and washed with water (3 *x* 30 ml). The organic phase was dried over anhydrous sodium sulfate, concentrated under reduced pressure and then purified by silica gel column chromatography eluting with petroleum ether/ethyl acetate (10/1–4/1, V/V) to give (I) as yellow solid. Crystals suitable for X-ray analysis were grown from a mixed solution of (I) in ethyl acetate and petroleum ether by slow evaporation at room temperature.

### Refinement

H atoms were positioned geometrically and refined using a riding model, with C—H = 0.93-0.97 Å and with *U*_iso_(H) = 1.2*U*_eq_(C).

### Crystal data

**Table:**

C_18_H_11_BrN_2_O_2_	*Z* = 4
*M**_r_* = 367.20	*F*(000) = 736
Triclinic, *P*1	*D*_x_ = 1.608 Mg m^−^^3^
Hall symbol: -P 1	Mo *K*α radiation, λ = 0.71073 Å
*a* = 10.460 (3) Å	Cell parameters from 1880 reflections
*b* = 11.816 (3) Å	θ = 2.4–23.5°
*c* = 13.561 (3) Å	µ = 2.72 mm^−^^1^
α = 75.128 (4)°	*T* = 296 K
β = 75.725 (4)°	Block, yellow
γ = 72.298 (4)°	0.26 × 0.24 × 0.18 mm
*V* = 1517.2 (7) Å^3^	

### Data collection

**Table:**

Bruker APEXII CCD diffractometer	5901 independent reflections
Radiation source: fine-focus sealed tube	3709 reflections with *I* > 2σ(*I*)
Graphite monochromator	*R*_int_ = 0.023
φ and ω scans	θ_max_ = 26.0°, θ_min_ = 1.6°
Absorption correction: multi-scan (*SADABS*; Sheldrick, 1996)	*h* = −8→12
*T*_min_ = 0.538, *T*_max_ = 0.640	*k* = −12→14
8441 measured reflections	*l* = −14→16

### Refinement

**Table:**

Refinement on *F*^2^	Primary atom site location: structure-invariant direct methods
Least-squares matrix: full	Secondary atom site location: difference Fourier map
*R*[*F*^2^ > 2σ(*F*^2^)] = 0.067	Hydrogen site location: inferred from neighbouring sites
*wR*(*F*^2^) = 0.189	H-atom parameters constrained
*S* = 1.04	*w* = 1/[σ^2^(*F*_o_^2^) + (0.0813*P*)^2^ + 2.8703*P*] where *P* = (*F*_o_^2^ + 2*F*_c_^2^)/3
5901 reflections	(Δ/σ)_max_ < 0.001
425 parameters	Δρ_max_ = 2.20 e Å^−^^3^
1 restraint	Δρ_min_ = −0.74 e Å^−^^3^

### Special details

**Table:**

Geometry. All e.s.d.'s (except the e.s.d. in the dihedral angle between two l.s. planes) are estimated using the full covariance matrix. The cell e.s.d.'s are taken into account individually in the estimation of e.s.d.'s in distances, angles and torsion angles; correlations between e.s.d.'s in cell parameters are only used when they are defined by crystal symmetry. An approximate (isotropic) treatment of cell e.s.d.'s is used for estimating e.s.d.'s involving l.s. planes.
Refinement. Refinement of *F*^2^ against ALL reflections. The weighted *R*-factor *wR* and goodness of fit *S* are based on *F*^2^, conventional *R*-factors *R* are based on *F*, with *F* set to zero for negative *F*^2^. The threshold expression of *F*^2^ > σ(*F*^2^) is used only for calculating *R*-factors(gt) *etc*. and is not relevant to the choice of reflections for refinement. *R*-factors based on *F*^2^ are statistically about twice as large as those based on *F*, and *R*- factors based on ALL data will be even larger.

### Fractional atomic coordinates and isotropic or equivalent isotropic displacement parameters (Å^2^)

**Table:**

	*x*	*y*	*z*	*U*_iso_*/*U*_eq_	Occ. (<1)
Br2	0.55459 (14)	0.50977 (11)	0.76724 (11)	0.0989 (5)	0.742 (2)
C19	0.6750 (6)	0.5968 (6)	0.7825 (5)	0.0631 (18)	
C27	0.4943 (7)	0.6809 (6)	0.9230 (5)	0.0630 (18)	
H27	0.4352	0.6387	0.9177	0.076*	0.742 (2)
Br2'	0.3819 (3)	0.5988 (3)	0.9048 (3)	0.0711 (11)	0.258 (2)
H19'	0.6195	0.5535	0.7732	0.076*	0.258 (2)
O3	1.0432 (4)	0.7900 (4)	0.7554 (3)	0.0576 (11)	
O4	0.7320 (4)	0.9391 (4)	1.0192 (3)	0.0579 (11)	
N3	0.8868 (4)	0.8680 (4)	0.8839 (3)	0.0391 (10)	
N4	0.9653 (5)	0.9410 (4)	0.8921 (3)	0.0429 (11)	
C20	0.7978 (8)	0.5865 (6)	0.7231 (5)	0.0658 (18)	
H20A	0.8266	0.5359	0.6751	0.079*	
C21	0.8830 (7)	0.6511 (6)	0.7325 (5)	0.0554 (16)	
H21A	0.9684	0.6451	0.6899	0.067*	
C22	0.8417 (6)	0.7239 (5)	0.8044 (4)	0.0440 (13)	
C23	0.7107 (5)	0.7359 (5)	0.8683 (4)	0.0398 (12)	
C24	0.6694 (5)	0.8110 (5)	0.9422 (4)	0.0384 (12)	
C25	0.5441 (6)	0.8219 (6)	1.0036 (4)	0.0512 (15)	
H25A	0.5160	0.8725	1.0516	0.061*	
C26	0.4565 (6)	0.7562 (6)	0.9943 (5)	0.0622 (18)	
H26A	0.3711	0.7637	1.0372	0.075*	
C28	0.6227 (6)	0.6695 (5)	0.8594 (5)	0.0506 (15)	
C29	0.9338 (6)	0.7938 (5)	0.8102 (4)	0.0415 (13)	
C30	0.7610 (5)	0.8777 (5)	0.9544 (4)	0.0416 (13)	
C31	1.0955 (6)	0.8715 (6)	0.9279 (5)	0.0524 (15)	
H31A	1.1567	0.8304	0.8741	0.063*	
H31B	1.1389	0.9269	0.9406	0.063*	
C32	1.0719 (6)	0.7828 (6)	1.0224 (5)	0.0516 (15)	
C33	1.0549 (7)	0.7110 (6)	1.0983 (5)	0.0650 (18)	
H33	1.0414	0.6537	1.1587	0.078*	
C34	0.9868 (6)	1.0320 (5)	0.7990 (5)	0.0529 (15)	
H34A	1.0319	1.0855	0.8126	0.063*	
H34B	1.0468	0.9922	0.7440	0.063*	
C35	0.8608 (7)	1.1035 (6)	0.7643 (5)	0.0513 (15)	
C36	0.7616 (8)	1.1615 (7)	0.7340 (6)	0.073 (2)	
H36	0.6821	1.2079	0.7098	0.088*	
Br1	0.59705 (8)	0.00106 (6)	0.23760 (5)	0.0651 (3)	
O1	0.5025 (4)	0.3271 (4)	0.6119 (4)	0.0619 (12)	
O2	0.0699 (4)	0.2945 (4)	0.6614 (3)	0.0534 (10)	
N1	0.2845 (4)	0.3213 (4)	0.6295 (3)	0.0384 (10)	
N2	0.2348 (5)	0.4032 (4)	0.6985 (3)	0.0411 (10)	
C1	0.5383 (7)	0.0905 (5)	0.3449 (4)	0.0507 (15)	
C2	0.6289 (6)	0.1396 (6)	0.3650 (4)	0.0513 (15)	
H2A	0.7169	0.1289	0.3260	0.062*	
C3	0.5900 (6)	0.2068 (5)	0.4446 (5)	0.0526 (15)	
H3A	0.6525	0.2389	0.4593	0.063*	
C4	0.4603 (6)	0.2243 (5)	0.4999 (4)	0.0431 (13)	
C5	0.3643 (6)	0.1758 (4)	0.4791 (4)	0.0412 (13)	
C6	0.2311 (6)	0.1948 (5)	0.5347 (4)	0.0417 (13)	
C7	0.1384 (6)	0.1445 (5)	0.5174 (4)	0.0500 (14)	
H7A	0.0501	0.1577	0.5557	0.060*	
C8	0.1779 (7)	0.0727 (6)	0.4415 (5)	0.0566 (16)	
H8A	0.1162	0.0370	0.4304	0.068*	
C9	0.3051 (7)	0.0557 (5)	0.3849 (4)	0.0536 (15)	
H9A	0.3286	0.0097	0.3338	0.064*	
C10	0.4030 (6)	0.1046 (5)	0.4001 (4)	0.0470 (14)	
C11	0.1848 (6)	0.2737 (5)	0.6133 (4)	0.0405 (13)	
C12	0.4232 (6)	0.2937 (5)	0.5833 (4)	0.0460 (13)	
C13	0.2805 (6)	0.3486 (5)	0.7979 (4)	0.0514 (15)	
H13A	0.2603	0.2703	0.8244	0.062*	
H13B	0.3784	0.3363	0.7882	0.062*	
C14	0.2116 (7)	0.4275 (6)	0.8720 (5)	0.0590 (16)	
C15	0.1620 (9)	0.4915 (8)	0.9295 (6)	0.091 (3)	
H15	0.1218	0.5435	0.9762	0.109*	
C16	0.2598 (6)	0.5219 (5)	0.6495 (5)	0.0499 (14)	
H16A	0.2196	0.5768	0.6977	0.060*	
H16B	0.3573	0.5144	0.6322	0.060*	
C17	0.2019 (6)	0.5705 (5)	0.5570 (5)	0.0508 (15)	
C18	0.1566 (8)	0.6073 (6)	0.4818 (6)	0.073 (2)	
H18	0.1203	0.6370	0.4213	0.088*	

### Atomic displacement parameters (Å^2^)

**Table:**

	*U*^11^	*U*^22^	*U*^33^	*U*^12^	*U*^13^	*U*^23^
Br2	0.1274 (11)	0.0833 (8)	0.1227 (10)	−0.0587 (8)	−0.0590 (8)	−0.0111 (7)
C19	0.090 (5)	0.042 (4)	0.072 (4)	−0.024 (3)	−0.040 (4)	−0.004 (3)
C27	0.062 (4)	0.057 (4)	0.073 (4)	−0.021 (3)	−0.025 (4)	0.000 (4)
Br2'	0.0594 (18)	0.0600 (18)	0.103 (2)	−0.0243 (13)	−0.0217 (15)	−0.0145 (15)
C19'	0.090 (5)	0.042 (4)	0.072 (4)	−0.024 (3)	−0.040 (4)	−0.004 (3)
C27'	0.062 (4)	0.057 (4)	0.073 (4)	−0.021 (3)	−0.025 (4)	0.000 (4)
O3	0.045 (2)	0.071 (3)	0.062 (3)	−0.021 (2)	0.011 (2)	−0.034 (2)
O4	0.056 (3)	0.072 (3)	0.053 (2)	−0.019 (2)	0.008 (2)	−0.038 (2)
N3	0.040 (3)	0.043 (3)	0.037 (2)	−0.012 (2)	−0.0010 (19)	−0.0156 (19)
N4	0.046 (3)	0.046 (3)	0.041 (2)	−0.019 (2)	−0.008 (2)	−0.010 (2)
C20	0.075 (5)	0.059 (4)	0.072 (4)	−0.016 (4)	−0.016 (4)	−0.026 (4)
C21	0.060 (4)	0.056 (4)	0.051 (3)	−0.009 (3)	−0.010 (3)	−0.018 (3)
C22	0.054 (4)	0.035 (3)	0.042 (3)	−0.010 (3)	−0.007 (3)	−0.010 (2)
C23	0.041 (3)	0.036 (3)	0.042 (3)	−0.012 (2)	−0.015 (2)	0.002 (2)
C24	0.040 (3)	0.037 (3)	0.035 (3)	−0.007 (2)	−0.007 (2)	−0.003 (2)
C25	0.044 (4)	0.055 (4)	0.048 (3)	−0.011 (3)	−0.009 (3)	−0.002 (3)
C26	0.033 (3)	0.071 (5)	0.071 (4)	−0.019 (3)	−0.009 (3)	0.011 (4)
C28	0.058 (4)	0.042 (3)	0.055 (3)	−0.016 (3)	−0.024 (3)	0.003 (3)
C29	0.040 (3)	0.043 (3)	0.039 (3)	−0.008 (2)	−0.005 (3)	−0.009 (2)
C30	0.045 (3)	0.044 (3)	0.035 (3)	−0.007 (2)	−0.008 (2)	−0.010 (2)
C31	0.055 (4)	0.059 (4)	0.050 (3)	−0.022 (3)	−0.011 (3)	−0.014 (3)
C32	0.055 (4)	0.057 (4)	0.048 (3)	−0.013 (3)	−0.012 (3)	−0.019 (3)
C33	0.080 (5)	0.064 (4)	0.051 (4)	−0.017 (4)	−0.011 (3)	−0.016 (3)
C34	0.058 (4)	0.048 (4)	0.054 (3)	−0.018 (3)	−0.002 (3)	−0.014 (3)
C35	0.056 (4)	0.049 (4)	0.050 (3)	−0.017 (3)	−0.012 (3)	−0.006 (3)
C36	0.072 (5)	0.067 (5)	0.081 (5)	−0.014 (4)	−0.024 (4)	−0.011 (4)
Br1	0.0852 (5)	0.0556 (4)	0.0410 (3)	−0.0003 (3)	−0.0023 (3)	−0.0167 (3)
O1	0.047 (3)	0.075 (3)	0.073 (3)	−0.019 (2)	−0.005 (2)	−0.031 (2)
O2	0.044 (2)	0.075 (3)	0.046 (2)	−0.018 (2)	−0.0006 (19)	−0.024 (2)
N1	0.035 (2)	0.038 (2)	0.038 (2)	−0.0026 (19)	−0.0018 (19)	−0.0125 (19)
N2	0.050 (3)	0.036 (2)	0.038 (2)	−0.007 (2)	−0.011 (2)	−0.0098 (19)
C1	0.063 (4)	0.041 (3)	0.034 (3)	−0.001 (3)	−0.004 (3)	−0.005 (2)
C2	0.041 (3)	0.060 (4)	0.042 (3)	−0.007 (3)	0.006 (3)	−0.011 (3)
C3	0.053 (4)	0.045 (3)	0.054 (3)	−0.011 (3)	−0.003 (3)	−0.008 (3)
C4	0.043 (3)	0.037 (3)	0.039 (3)	−0.001 (2)	−0.004 (2)	−0.004 (2)
C5	0.056 (4)	0.030 (3)	0.030 (2)	−0.004 (2)	−0.011 (2)	0.001 (2)
C6	0.050 (3)	0.039 (3)	0.032 (3)	−0.008 (2)	−0.008 (2)	−0.005 (2)
C7	0.055 (4)	0.051 (4)	0.046 (3)	−0.012 (3)	−0.011 (3)	−0.015 (3)
C8	0.066 (4)	0.057 (4)	0.056 (4)	−0.020 (3)	−0.018 (3)	−0.015 (3)
C9	0.071 (5)	0.046 (4)	0.044 (3)	−0.012 (3)	−0.011 (3)	−0.013 (3)
C10	0.058 (4)	0.037 (3)	0.033 (3)	−0.002 (3)	−0.007 (3)	0.001 (2)
C11	0.050 (4)	0.040 (3)	0.029 (2)	−0.009 (3)	−0.009 (3)	−0.003 (2)
C12	0.046 (4)	0.041 (3)	0.048 (3)	−0.008 (3)	−0.012 (3)	−0.006 (3)
C13	0.061 (4)	0.052 (4)	0.040 (3)	−0.010 (3)	−0.015 (3)	−0.006 (3)
C14	0.069 (4)	0.061 (4)	0.048 (3)	−0.010 (3)	−0.012 (3)	−0.020 (3)
C15	0.131 (7)	0.081 (6)	0.060 (4)	−0.008 (5)	−0.021 (5)	−0.032 (4)
C16	0.050 (3)	0.040 (3)	0.059 (3)	−0.003 (3)	−0.018 (3)	−0.010 (3)
C17	0.061 (4)	0.038 (3)	0.047 (3)	−0.002 (3)	−0.010 (3)	−0.009 (3)
C18	0.089 (5)	0.054 (4)	0.066 (4)	0.002 (4)	−0.020 (4)	−0.012 (3)

### Geometric parameters (Å, º)

**Table:**

Br2—C19	1.927 (6)	Br1—C1	1.888 (6)
C19—C20	1.326 (9)	O1—C12	1.197 (7)
C19—C28	1.421 (9)	O2—C11	1.202 (6)
C27—C26	1.386 (9)	N1—C12	1.405 (7)
C27—C28	1.394 (9)	N1—C11	1.409 (7)
C27—H27	0.9300	N1—N2	1.415 (6)
O3—C29	1.196 (6)	N2—C16	1.462 (7)
O4—C30	1.205 (6)	N2—C13	1.465 (7)
N3—C29	1.397 (7)	C1—C2	1.361 (9)
N3—N4	1.399 (6)	C1—C10	1.411 (8)
N3—C30	1.416 (7)	C2—C3	1.410 (8)
N4—C34	1.452 (7)	C2—H2A	0.9300
N4—C31	1.486 (7)	C3—C4	1.361 (8)
C20—C21	1.383 (9)	C3—H3A	0.9300
C20—H20A	0.9300	C4—C5	1.409 (8)
C21—C22	1.369 (8)	C4—C12	1.473 (8)
C21—H21A	0.9300	C5—C6	1.392 (8)
C22—C23	1.417 (8)	C5—C10	1.437 (8)
C22—C29	1.474 (8)	C6—C7	1.372 (8)
C23—C24	1.411 (7)	C6—C11	1.491 (7)
C23—C28	1.421 (8)	C7—C8	1.405 (8)
C24—C25	1.357 (8)	C7—H7A	0.9300
C24—C30	1.473 (8)	C8—C9	1.346 (9)
C25—C26	1.414 (9)	C8—H8A	0.9300
C25—H25A	0.9300	C9—C10	1.396 (9)
C26—H26A	0.9300	C9—H9A	0.9300
C31—C32	1.454 (9)	C13—C14	1.453 (8)
C31—H31A	0.9700	C13—H13A	0.9700
C31—H31B	0.9700	C13—H13B	0.9700
C32—C33	1.168 (9)	C14—C15	1.147 (9)
C33—H33	0.9300	C15—H15	0.9300
C34—C35	1.449 (9)	C16—C17	1.436 (8)
C34—H34A	0.9700	C16—H16A	0.9700
C34—H34B	0.9700	C16—H16B	0.9700
C35—C36	1.161 (9)	C17—C18	1.157 (9)
C36—H36	0.9300	C18—H18	0.9300
			
C20—C19—C28	124.4 (6)	C12—N1—C11	125.4 (4)
C20—C19—Br2	120.0 (5)	C12—N1—N2	120.1 (4)
C28—C19—Br2	115.6 (5)	C11—N1—N2	114.5 (4)
C26—C27—C28	118.8 (6)	N1—N2—C16	111.9 (4)
C26—C27—H27	120.6	N1—N2—C13	111.2 (4)
C28—C27—H27	120.6	C16—N2—C13	115.4 (4)
C29—N3—N4	120.3 (4)	C2—C1—C10	122.8 (5)
C29—N3—C30	125.2 (5)	C2—C1—Br1	118.2 (4)
N4—N3—C30	114.5 (4)	C10—C1—Br1	119.0 (5)
N3—N4—C34	113.2 (4)	C1—C2—C3	120.3 (5)
N3—N4—C31	113.7 (4)	C1—C2—H2A	119.9
C34—N4—C31	111.7 (4)	C3—C2—H2A	119.9
C19—C20—C21	119.9 (6)	C4—C3—C2	119.5 (6)
C19—C20—H20A	120.0	C4—C3—H3A	120.2
C21—C20—H20A	120.0	C2—C3—H3A	120.2
C22—C21—C20	120.1 (6)	C3—C4—C5	121.1 (5)
C22—C21—H21A	120.0	C3—C4—C12	118.2 (6)
C20—C21—H21A	120.0	C5—C4—C12	120.7 (5)
C21—C22—C23	120.6 (6)	C6—C5—C4	121.2 (5)
C21—C22—C29	118.5 (5)	C6—C5—C10	118.6 (5)
C23—C22—C29	120.8 (5)	C4—C5—C10	120.2 (5)
C24—C23—C22	120.1 (5)	C7—C6—C5	121.5 (5)
C24—C23—C28	120.2 (5)	C7—C6—C11	118.2 (5)
C22—C23—C28	119.7 (5)	C5—C6—C11	120.2 (5)
C25—C24—C23	119.9 (5)	C6—C7—C8	119.5 (6)
C25—C24—C30	119.2 (5)	C6—C7—H7A	120.2
C23—C24—C30	120.9 (5)	C8—C7—H7A	120.2
C24—C25—C26	119.7 (6)	C9—C8—C7	120.0 (6)
C24—C25—H25A	120.1	C9—C8—H8A	120.0
C26—C25—H25A	120.1	C7—C8—H8A	120.0
C27—C26—C25	121.8 (6)	C8—C9—C10	122.5 (6)
C27—C26—H26A	119.1	C8—C9—H9A	118.8
C25—C26—H26A	119.1	C10—C9—H9A	118.8
C27—C28—C19	125.2 (6)	C9—C10—C1	126.1 (6)
C27—C28—C23	119.5 (6)	C9—C10—C5	117.8 (5)
C19—C28—C23	115.3 (6)	C1—C10—C5	116.1 (6)
O3—C29—N3	120.2 (5)	O2—C11—N1	121.6 (5)
O3—C29—C22	123.2 (5)	O2—C11—C6	122.4 (5)
N3—C29—C22	116.6 (5)	N1—C11—C6	116.0 (5)
O4—C30—N3	120.6 (5)	O1—C12—N1	119.9 (5)
O4—C30—C24	123.1 (5)	O1—C12—C4	124.3 (5)
N3—C30—C24	116.3 (4)	N1—C12—C4	115.8 (5)
C32—C31—N4	111.0 (5)	C14—C13—N2	109.8 (5)
C32—C31—H31A	109.4	C14—C13—H13A	109.7
N4—C31—H31A	109.4	N2—C13—H13A	109.7
C32—C31—H31B	109.4	C14—C13—H13B	109.7
N4—C31—H31B	109.4	N2—C13—H13B	109.7
H31A—C31—H31B	108.0	H13A—C13—H13B	108.2
C33—C32—C31	179.0 (7)	C15—C14—C13	177.1 (9)
C32—C33—H33	180.0	C14—C15—H15	180.0
C35—C34—N4	112.8 (5)	C17—C16—N2	110.5 (5)
C35—C34—H34A	109.0	C17—C16—H16A	109.5
N4—C34—H34A	109.0	N2—C16—H16A	109.5
C35—C34—H34B	109.0	C17—C16—H16B	109.5
N4—C34—H34B	109.0	N2—C16—H16B	109.5
H34A—C34—H34B	107.8	H16A—C16—H16B	108.1
C36—C35—C34	178.3 (7)	C18—C17—C16	178.6 (7)
C35—C36—H36	180.0	C17—C18—H18	180.0
			
C29—N3—N4—C34	62.9 (6)	C12—N1—N2—C16	58.3 (6)
C30—N3—N4—C34	−116.9 (5)	C11—N1—N2—C16	−121.7 (5)
C29—N3—N4—C31	−66.0 (6)	C12—N1—N2—C13	−72.5 (6)
C30—N3—N4—C31	114.2 (5)	C11—N1—N2—C13	107.6 (5)
C28—C19—C20—C21	−1.4 (10)	C10—C1—C2—C3	0.8 (9)
Br2—C19—C20—C21	178.6 (5)	Br1—C1—C2—C3	−179.7 (4)
C19—C20—C21—C22	1.2 (10)	C1—C2—C3—C4	−1.4 (9)
C20—C21—C22—C23	−1.3 (9)	C2—C3—C4—C5	0.3 (8)
C20—C21—C22—C29	−178.4 (5)	C2—C3—C4—C12	179.4 (5)
C21—C22—C23—C24	179.9 (5)	C3—C4—C5—C6	−179.2 (5)
C29—C22—C23—C24	−3.1 (8)	C12—C4—C5—C6	1.8 (7)
C21—C22—C23—C28	1.5 (8)	C3—C4—C5—C10	1.3 (8)
C29—C22—C23—C28	178.5 (5)	C12—C4—C5—C10	−177.8 (5)
C22—C23—C24—C25	−179.8 (5)	C4—C5—C6—C7	−177.7 (5)
C28—C23—C24—C25	−1.4 (7)	C10—C5—C6—C7	1.8 (7)
C22—C23—C24—C30	−0.1 (7)	C4—C5—C6—C11	3.8 (7)
C28—C23—C24—C30	178.3 (5)	C10—C5—C6—C11	−176.7 (4)
C23—C24—C25—C26	1.1 (8)	C5—C6—C7—C8	−0.5 (8)
C30—C24—C25—C26	−178.5 (5)	C11—C6—C7—C8	178.1 (5)
C28—C27—C26—C25	0.4 (9)	C6—C7—C8—C9	−1.3 (9)
C24—C25—C26—C27	−0.6 (9)	C7—C8—C9—C10	1.7 (9)
C26—C27—C28—C19	−179.5 (6)	C8—C9—C10—C1	179.6 (6)
C26—C27—C28—C23	−0.6 (9)	C8—C9—C10—C5	−0.3 (8)
C20—C19—C28—C27	−179.6 (6)	C2—C1—C10—C9	−179.1 (6)
Br2—C19—C28—C27	0.5 (8)	Br1—C1—C10—C9	1.5 (8)
C20—C19—C28—C23	1.5 (9)	C2—C1—C10—C5	0.8 (8)
Br2—C19—C28—C23	−178.4 (4)	Br1—C1—C10—C5	−178.7 (4)
C24—C23—C28—C27	1.1 (8)	C6—C5—C10—C9	−1.5 (7)
C22—C23—C28—C27	179.5 (5)	C4—C5—C10—C9	178.1 (5)
C24—C23—C28—C19	−179.9 (5)	C6—C5—C10—C1	178.7 (5)
C22—C23—C28—C19	−1.5 (7)	C4—C5—C10—C1	−1.8 (7)
N4—N3—C29—O3	1.8 (8)	C12—N1—C11—O2	174.4 (5)
C30—N3—C29—O3	−178.4 (5)	N2—N1—C11—O2	−5.6 (7)
N4—N3—C29—C22	−177.6 (4)	C12—N1—C11—C6	−4.6 (7)
C30—N3—C29—C22	2.2 (7)	N2—N1—C11—C6	175.3 (4)
C21—C22—C29—O3	−0.2 (8)	C7—C6—C11—O2	−0.2 (8)
C23—C22—C29—O3	−177.3 (5)	C5—C6—C11—O2	178.4 (5)
C21—C22—C29—N3	179.2 (5)	C7—C6—C11—N1	178.9 (5)
C23—C22—C29—N3	2.1 (7)	C5—C6—C11—N1	−2.5 (7)
C29—N3—C30—O4	175.9 (5)	C11—N1—C12—O1	−170.7 (5)
N4—N3—C30—O4	−4.4 (7)	N2—N1—C12—O1	9.4 (8)
C29—N3—C30—C24	−5.1 (7)	C11—N1—C12—C4	9.8 (7)
N4—N3—C30—C24	174.6 (4)	N2—N1—C12—C4	−170.1 (4)
C25—C24—C30—O4	2.6 (8)	C3—C4—C12—O1	−6.8 (9)
C23—C24—C30—O4	−177.0 (5)	C5—C4—C12—O1	172.3 (5)
C25—C24—C30—N3	−176.3 (5)	C3—C4—C12—N1	172.8 (5)
C23—C24—C30—N3	4.0 (7)	C5—C4—C12—N1	−8.2 (7)
N3—N4—C31—C32	−51.7 (6)	N1—N2—C13—C14	−171.5 (5)
C34—N4—C31—C32	178.6 (5)	C16—N2—C13—C14	59.6 (7)
N4—C31—C32—C33	176 (100)	N2—C13—C14—C15	−99 (15)
N3—N4—C34—C35	51.8 (6)	N1—N2—C16—C17	55.8 (6)
C31—N4—C34—C35	−178.3 (5)	C13—N2—C16—C17	−175.6 (5)
N4—C34—C35—C36	−157 (25)	N2—C16—C17—C18	−52 (32)
